# Effect of Endocrown Restorations with Different CAD/CAM Materials: 3D Finite Element and Weibull Analyses

**DOI:** 10.1155/2017/5638683

**Published:** 2017-09-28

**Authors:** Laden Gulec, Nuran Ulusoy

**Affiliations:** Department of Restorative Dentistry, School of Dentistry, Near East University, Northern Nicosia, Northern Cyprus, Mersin 10, Turkey

## Abstract

The aim of this study was to evaluate the effects of two endocrown designs and computer aided design/manufacturing (CAD/CAM) materials on stress distribution and failure probability of restorations applied to severely damaged endodontically treated maxillary first premolar tooth (MFP). Two types of designs without and with 3 mm intraradicular extensions, endocrown (E) and modified endocrown (ME), were modeled on a 3D Finite element (FE) model of the MFP. Vitablocks Mark II (VMII), Vita Enamic (VE), and Lava Ultimate (LU) CAD/CAM materials were used for each type of design. von Mises and maximum principle values were evaluated and the Weibull function was incorporated with FE analysis to calculate the long term failure probability. Regarding the stresses that occurred in enamel, for each group of material, ME restoration design transmitted less stress than endocrown. During normal occlusal function, the overall failure probability was minimum for ME with VMII. ME restoration design with VE was the best restorative option for premolar teeth with extensive loss of coronal structure under high occlusal loads. Therefore, ME design could be a favorable treatment option for MFPs with missing palatal cusp. Among the CAD/CAM materials tested, VMII and VE were found to be more tooth-friendly than LU.

## 1. Introduction

Restoration of endodontically treated (ET) teeth has been a challenging procedure in restorative dentistry because of their high risk for biomechanical failure [1–5]. Teeth are susceptible to fracture as a result of reduced water content and loss of structural integrity associated with deep dental caries, trauma, or restorative and endodontic procedures [1, 2, 4, 5]. To provide the best prognosis for longevity with respect to these teeth, clinician must minimize the risk of future tooth fracture by selecting a design and a material that suits best to maximize the function and appearance of tooth [6, 7].

With the development of adhesive dentistry and advent of reinforced-ceramic materials, restoration of teeth with extensive loss of coronal tissue became feasible by means of cuspal coverage restorations including endocrowns. Endocrowns, defined as “bonded overlay restorations,” are anchored to the internal portion of the pulp chamber and on the cavity margins in order to obtain macromechanical retention whereas micromechanical retention is provided by the use of adhesive cementation [8, 9]. For teeth that have extensive loss of sound tooth structure, the need for further intraradicular extensions might be a prerequisite [10].

Finite Element (FE) analysis has been a complementary tool in understanding the process of stress distribution providing information to describe how the design of restorations and restorative materials having widely different behavioral properties affect the teeth [11, 12]. Analysis based on the mathematical modeling that examines the deformations under load of a model consists of a mesh of elements with given mechanical properties [12]. Weibull analysis is a function usually has been used for calculating the probability for fracture in brittle materials. It is important in predicting cumulative failure probability at selected stress levels [1].

In addition to the high strength ceramic materials, polymer-infiltrated ceramic network material, nanoceramic and composite materials have been developed with improved mechanical properties to be used with computer aided design/manufacturing (CAD/CAM) technology adding an important dimension to restorations involving cusp coverage [10, 13, 14].

To date, there is no clear consensus in the literature which endocrown design with which CAD/CAM material is the more effective treatment option to restore ET two-rooted maxillary first premolar (MFP) tooth with extensive loss of tooth structure. Therefore, the aim of this study was to evaluate the effects of two different endocrown designs and different materials available for CAD/CAM systems on stress distribution and failure probability of MFP with missing palatal cusp by means of FE and Weibull analyses.

## 2. Materials and Methods

This 3-dimensional (3D) FE study was conducted using Rhinoceros 4.0 3D modeling software (McNeel North America, Seattle, WA, USA), VR Mesh studio meshing software (Virtual Gird Inc, Bellevue City, WA, USA), and Algor Fempro analysis program (ALGOR, Inc. Pittsburgh, PA, USA).

### 2.1. Solid and FE Model Design

The external shape of the solid model was obtained by scanning a plaster model of MFP with Smart Optics (Smart Optics Sensortechnik GmbH, Bochum, Germany) and morphology of the model was generated by Wheeler's atlas [15]. The solid models which consisted of MFP with 0.2 mm thick periodontal ligament, 0.2 mm thick lamina dura, and cortical and trabecular surrounding bone were generated. Cortical bone structure was constructed having 1.5 mm thickness. The structures were assumed to be linearly elastic, isotropic, and homogenous for simplification and to overcome computing difficulties [16].

**Figure 1:**
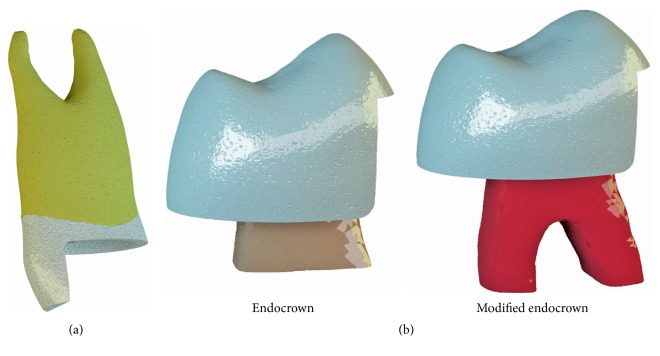
(a) Cavity design of endodontically treated maxillary first premolar. (b) Restoration designs used in this study.

A mesial-occlusal-distal-palatal (MODP) cavity was designed with 2.0 mm sound tissue above cementoenamel junction for the models. The cavity received a further 2.0 mm high reduction of the buccal cusp (Figure 1(a)) and was then restored by two different types of restorations (Figure 1(b)):

*(a) Endocrown (E)*. Macromechanical retention was provided by the internal portion of the pulp chamber for the endocrown design.

*(b) Modified Endocrown (ME)*. In addition to the pulp chamber, 3.0 mm intraradicular extensions were generated to both canals for macromechanical retention.

Three different CAD/CAM materials were used for each type of restoration design;

(*1) A Feldspathic Ceramic*. Vitablocks Mark II (VMII) (Vita Zahnfabrik, Bad Säckingen, Germany)

(*2) A Polymer-Infiltrated Hybrid Ceramic*. Vita Enamic (VE) (Vita Zahnfabrik, Bad Säckingen, Germany)

(*3) A Nanoceramic Resin*. Lava Ultimate (LU) (3M ESPE, Bad Seefeld, Germany).

Mechanical properties including Young's Modulus and Poisson's Ratio of the dental structures and materials simulated were determined from the literature [2, 17–20] and presented in Table 1. Young's Modulus is a measure of stiffness of an elastic material whereas Poisson's ratio is the ratio of the transverse strain (perpendicular to the applied load) to the axial strain (in the direction of the applied load) [16].

Bricks and tetrahedral solid elements with different number of elements and nodes were prepared to generate the models (Table 2).

**Figure 2:**
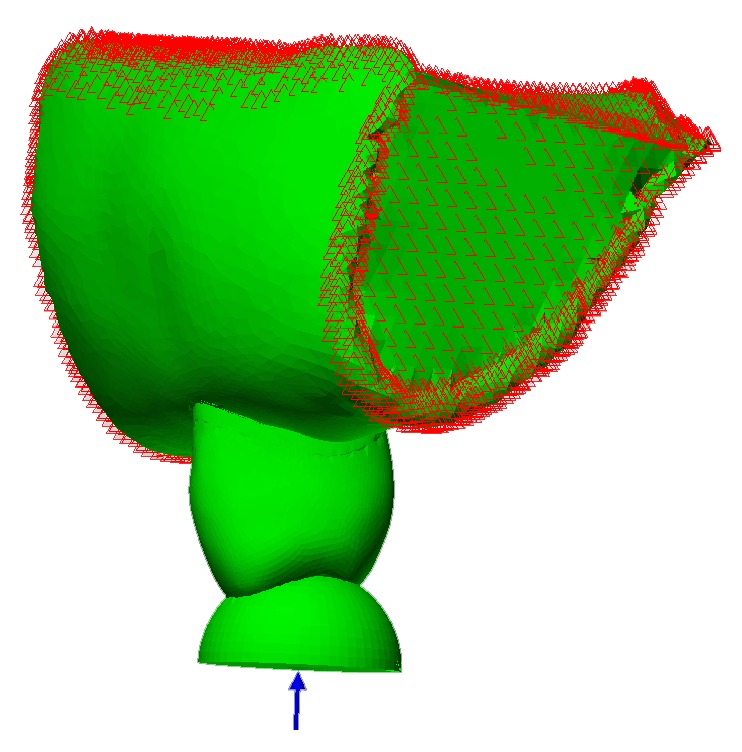
Spherical solid rigid material simulating the foodstuff which was 8.6 mm in diameter was loaded in the vertical direction to prevent localized contact.

A 100 N occlusal load was used to simulate foodstuff by a spherical solid rigid material (SSRM) (Figure 2). Since the FE models were linear, stresses for other loads (200 N–900 N; in 100 N increments) were calculated in proportion to the data in 100 N. To analyze stress distribution and location, all structures were isolated from the rest of the model. For all designs, von Mises and maximum principal stresses on the remaining enamel, remaining dentin, and restorative materials were evaluated in megapascals (MPa) separately.

### 2.2. Weibull Analysis

Weibull risk of rupture analysis was then used with the following equations in which the survival probability, *P*_*S*_, is given as follows [21, 22]:

(1) $$\begin{array}{c} P_{S}\left(\;\sigma \;\right)=\exp\left[\;-{\left(\;\frac{\sigma }{\sigma_{0}}\;\right)}^{m}\;\right], \end{array}$$

where *P*_*S*_ represents the survival probability of node at stress *σ* (for load *F*), *σ* represents the failure stress (maximum principal stress), *σ*_0_ represents the characteristic strength that is a normalized parameter corresponding with a stress level where 63% of the specimens fail, and *m* represents the Weibull modulus, that is, a parameter indicating the nature, severity, and spread of the defects [23]. When loaded, a restoration will survive until the risk of rupture reaches a critical value at any one of the multiple failure sources. Hence, for system of *n* = *i* sources, the overall survival probability, *P*_*S*_, is the product of the individual survival probabilities:

(2) $$\begin{array}{c} P_{S}=\underset{i}{\prod }P_{Si}, \end{array}$$

where *i* = 1,2, 3 in the case of the premolar MODP cavity with different restorations, because the stress concentration regions of enamel, dentin, and restorative material were observed at risk. Hence, the failure probability, *P*_*f*_, for the total systems is as follows:

(3) $$\begin{array}{c} P_{f}=1-\left(\;P_{S1}\times P_{S2}\times P_{S3}\;\right). \end{array}$$

Characteristic strengths and Weibull modulus of dental tissues and different materials were adopted for the calculation from the literature data [1, 18] (Table 1).

## 3. Results

### 3.1. Stress Distributions in Enamel

For von Mises, the highest stress values were observed in E2 (24.45 MPa). The lowest stress value was observed in ME1 with 9.66 MPa. The increasing stress values in endocrown model were as follows; VMII < LU < VE. Stress value of ME3 was 2.2 and 2 times higher than ME1 and ME2, respectively.

The maximum principle stress values revealed that E3 (15.88 MPa) had the highest stress value followed by ME3 (14.19 MPa) while ME1 had the lowest value with 5.78 MPa. In regard with the distribution pattern, it was observed that maximum stresses accumulated in cervical region of enamel for all models.

**Figure 3:**
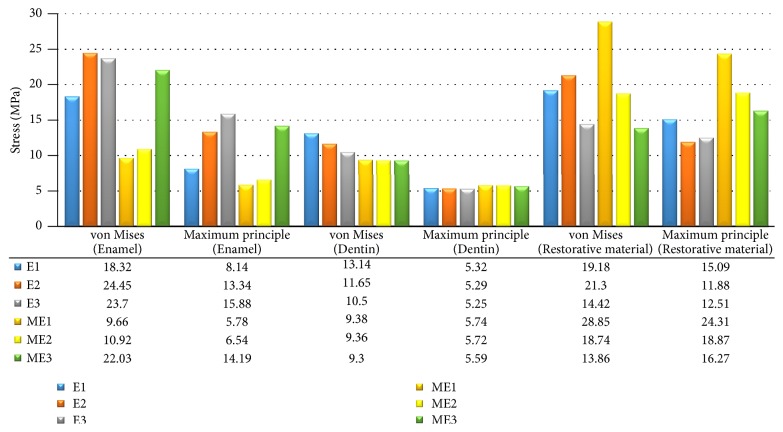
Maximum von Mises and maximum principle stress values which occurred in enamel, dentin, and restorative material. E1: endocrown with Vitablocks Mark II; E2: endocrown with Vita Enamic; E3: endocrown with Lava Ultimate; ME1: modified endocrown with Vitablocks Mark II; ME2: modified endocrown with Vita Enamic; ME3: modified endocrown with Lava Ultimate.

Stress values of maximum von Mises and maximum principle stresses are shown in Figure 3.

### 3.2. Stress Distributions in Dentin

For von Mises, E1 had the maximum stress value of 13.14 MPa. ME3 had the lowest stress value with 9.3 MPa. Stress value of E1 was 1.4 times greater than ME1. The increasing stress values of materials of restoration models were as follows: LU < VE < VMII.

Similar maximum principal stress values were observed between models (Figure 3) and distribution patterns showed that the highest maximum principle stress values were observed in the furcal region.

### 3.3. Stress Distributions in Restorative Materials

The analysis of von Mises stress values revealed that maximum stress concentrations were located at loading areas for all models. Both designs showed that palatinal aspect of extensions had intense stress accumulations besides occlusal loading area. ME1 had the greatest stress value among all models, while ME3 had the minimum. Endocrown design with VE had higher stress values than ME. Stresses seemed to be approximately similar between the two restoration designs with LU.

For maximum principle stress, the greatest stress value was observed in ME1 (24.31 MPa) and the lowest value was seen in E2 with 11.88 MPa. VMII showed the highest tension for both models.

**Figure 4:**
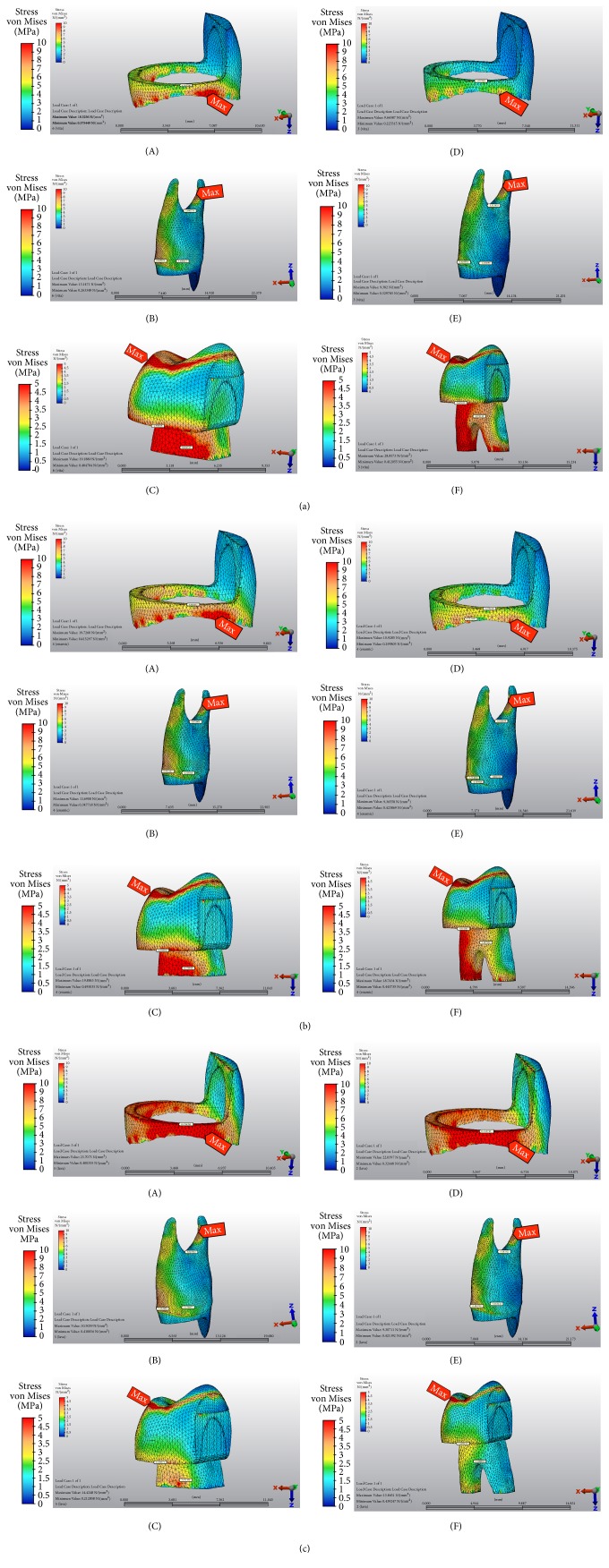
(a) von Mises stress distribution patterns of models with Vitablocks Mark II. First scale is related to (A) and (D); middle scale is related to (B) and (E); third scale is related to (C) and (F). The red indicators show the maximum value of stress distribution. (A) Enamel of endocrown, (B) dentin of endocrown, (C) restorative material of endocrown, (D) enamel of modified endocrown, (E) dentin of modified endocrown, and (F) restorative material of modified endocrown. (b) von Mises stress distribution patterns of models with Vita Enamic. First scale is related to (A) and (D); middle scale is related to (B) and (E); third scale is related to (C) and (F) The red indicators show the maximum value of stress distribution. (A) Enamel of endocrown, (B) dentin of endocrown, (C) restorative material of endocrown, (D) enamel of modified endocrown, (E) dentin of modified endocrown, and (F) restorative material of modified endocrown. (c) von Mises stress distribution patterns of models with Lava Ultimate. First scale is related to (A) and (D); middle scale is related to (B) and (E); third scale is related to (C) and (F). The red indicators show the maximum value of stress distribution. (A) Enamel of endocrown, (B) dentin of endocrown, (C) restorative material of endocrown, (D) enamel of modified endocrown, (E) dentin of modified endocrown, and (F) restorative material of modified endocrown.

Maximum von Mises and maximum principle stress values of models are shown in Figure 3.

von Mises stress distribution patterns of models are shown in Figures 4(a)–4(c).

### 3.4. Weibull Risk of Rupture Analysis

**Figure 5:**
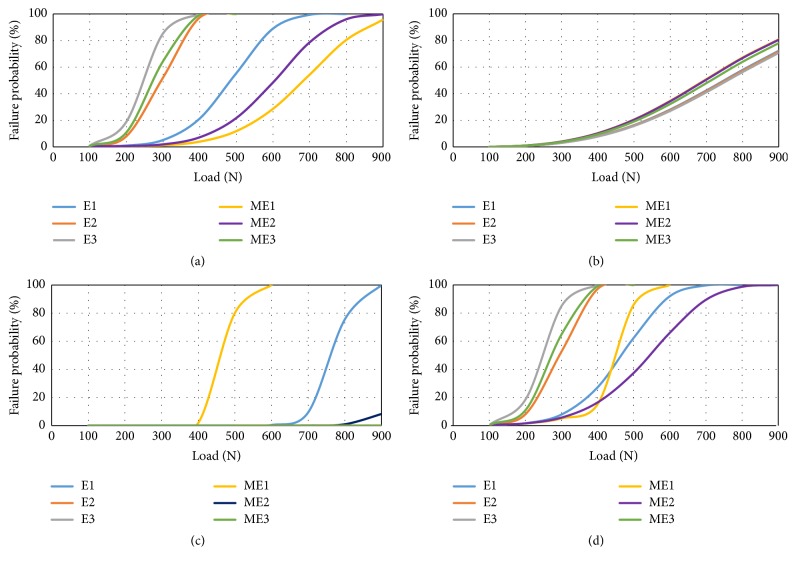
Failure probability versus load curves of models according to Weibull risk of rupture analysis (a) enamel, (b) dentin, (c) restorative material, and (d) overall failure probability. E1: endocrown with Vitablocks Mark II; E2: endocrown with Vita Enamic; E3: endocrown with Lava Ultimate; ME1: modified endocrown with Vitablocks Mark II; ME2: modified endocrown with Vita Enamic; ME3: modified endocrown with Lava Ultimate.

The failure probabilities of individual enamel, dentin, restorative material, and overall failure probability are shown in Figures 5(a)–5(d). In case of enamel, models with LU showed higher risk of failure (Figure 5(a)). Risk of failure distributions of models were similar in dentin (Figure 5(b)). When evaluated from the material standpoint, VMII was the only material showing failure (Figure 5(c)).

The overall failure probabilities (Figure 5(d)) of ME1, ME2, and E1 were 4%, 5%, and 7% under normal occlusal loads, respectively. For high occlusal loads, ME2 was the most successful model. LU was found to be the material having the highest risk of rupture regardless from the type of the restoration.

## 4. Discussion

The goal of restorative dentistry is to replace the lost dental tissue with appropriate methods using materials whose structure and physical properties are similar to a natural tooth. As the structural strength of the tooth with extensive loss of tooth structure restored with conventional restorations is poor, endocrowns became an alternative option for the treatment of these teeth [1, 24, 25].

Chang et al. [26] reported that endocrown technique was first described by Pissis who suggested a 5 mm depth central retention cavity for the first maxillary premolars although dimensions for the preparation of central retention cavity were not clearly determined. Chang et al. [26] used a central retention cavity of 5 mm preparation depth in their research for a single rooted maxillary premolar. In this study, we aimed to evaluate the stress distribution and failure probability of a modified endocrown model for a two-rooted maxillary premolar having a definitive radicular internal support besides the pulp chamber support. The level of the pulp chamber floor is modeled at the level of cementoenamel junction according to Krasner and Rankow's [27] findings. For this reason, a 2 mm deep pulp chamber cavity was designed and 3 mm intraradicular retentions were modeled in this study to obtain a 5 mm retention area for modified endocrown model.

Taking into consideration the outcome of an early study which showed that stresses found in dental tissues with 1 mm thick replacement were greater than stresses in 1.5 mm and 2 mm designs [6], reduction in buccal cusp height was chosen to be 2 mm in this study.

MFP model was used in this study because these teeth are more prone to have fracture especially on functional cusps related to their unfavorable anatomic shape, crown volume, and crown/root portion [7, 28]. In addition, removal of tooth structure for endodontic and restorative procedures results in an increase in cuspal deflection and tooth fragility under occlusal forces [24].

In the literature, the luting cement thickness was accepted as a part of dental tissues and stresses were not evaluated for cement because it was too thin to adequately model in finite element simulation [29, 30]. Furthermore, in a study, no statistical differences in stresses were found between cement thickness varying from 50 to 150 *μ*m on the remaining enamel and dentin for ceramic systems [6]. For this reason, in the present study, the thin luting cement thickness was neglected.

Previous research was conducted using oblique or vertical loads during the application of load to FE models [4, 11, 31]. As teeth are subjected to functional and parafunctional forces of varying magnitudes and directions during chewing, when the load is applied from one point, it cannot simulate the oral environment. For this reason, 100 N occlusal force was applied in the vertical direction by a SSRM to simulate foodstuff in order to better simulate the oral environment in this study. According to the size of the tooth in the models, 8.6 mm diameter was chosen for SSRM in order to prevent localized contact.

Stress analysis methods are used to evaluate the various stresses generated on the oral tissues in predicting the clinical performance of restorative materials. FE analysis is one of the best methods to simulate the oral environment* in vitro *[32]. In our study, FE method was chosen to evaluate the effects of stress distribution of MFP restored with different endocrown designs and CAD/CAM materials. FE analysis data is expressed as tensile, compressive, shear, or von Mises stresses distributed in the related structures investigated. von Mises stresses are a combination of tensile, compressive, and shear stresses that depend on the entire stress field. von Mises stresses are indicators of the possible damage occurrence and maximum principle stress are accepted as a suitable index to judge the materials failure that is assumed to be brittle [1, 33]. Regarding the stresses that occurred in enamel, for each group of material, ME restoration design transmitted less stress highlighting that it is a more tooth-friendly design than endocrown. Additionally, restorations with VMII had the lowest von Mises and maximum principle values in enamel, compared to other materials for both designs pointing out that materials with high elastic modulus are capable of protecting sound enamel tissue by transmitting less stress. These findings are consistent with the results of the study which defended that materials with low elastic moduli transferred more stress to dental tissues [11].

Regarding the stresses that occurred in restorative materials, maximum principle stress values were higher for the modified endocrown restoration design. Since the volume of material used for ME design is more, the stress value that is absorbed by the material is relatively more. When the volume of the material used for the restoration increases, the material itself is adversely affected but the stress transmitted to the dental tissues is reduced.

Clinically, the normal biting force is 222–445 N (average, 322.5 N) for the maxillary premolar area. During clenching, the occlusal force has been observed to be as high as 520–800 N (average 660 N) [34, 35]. The overall failure probabilities of the models in this study showed that although ME with VMII was very successful under normal occlusal functional loads it could not resist high occlusal loads. On the other hand, modified endocrown with VE showed better resistance than the other materials and the failure was observed under 900 N occlusal load.

There have been different Weibull moduli and characteristic strength values of CAD/CAM materials used in the literature due to the material, fabrication process, stress configuration, test specimen size, or differences in the number of specimens [36–38]. The Weibull moduli of CAD/CAM materials used in this study showed high values reflecting the advanced technological stage in the fabrication and processing of presintered of fully sintered ceramic-based blocks for dental restorations. Gonzaga et al. [23] stated that materials with lower characteristic strengths and higher *m* values can be chosen for restorations under low stress loads. In this study we got a consistent result with this finding that VMII having highest *m* value and lowest characteristic strength did not show stress accumulation under normal occlusal loads. On the other hand, it was the only material that showed failure in ME and E restoration designs under 600 N and 900 N occlusal loads, respectively. Wendler et al. [18] reported that proper analyses are mandatory on the basis of Weibull theory for brittle fracture to prevent the discrepancies from variety of the results. We agree with Wendler et al. [18] and suggest that more studies are required with different Weibull moduli and characteristic strength values of three CAD/CAM materials to analyze the effect of the variety of the results.

Although FE analysis has been suggested as a reliable technique simulating the* in vivo* conditions for the analysis of stress distributions, it may not always reflect the oral conditions along with complex loads. The Weibull function and FE analysis are combined in this study to provide an alternative method for predicting cumulative failure probability to longevity for restorations. Therefore, a more realistic result is obtained by predicting the fatigue lifetime besides stress distributions of restorations. However, there have been some limitations in this study. The theoretical assumptions such as loading conditions, accepting resin cement as a part of dental tissues, and material properties (linearly elastic, homogeneous, and isotropic) could bias the results. As the changes in the Weibull moduli would affect the results and hence the decision, the Weibull data taken from the studies in the literature should be cautiously evaluated. In order to eliminate the disadvantages of assumptions and differences in values of parameters and get a better insight into the biomechanical aspects and estimation risk of the endodontically treated MFP, behavior of different endocrown designs and materials, in the treatment of cuspal fracture of maxillary first premolars should be evaluated with laboratory experiments and long term clinical trials.

## 5. Conclusions

The results of this FE and Weibull analysis study in regard to the limitations suggested the following:

1. In regard to the restoration design, the modified endocrown design with intraradicular extensions protected the remaining tooth structures better than endocrown design.
2. On the effect of restorative materials tested, VMII was only successful in protecting tooth structures under normal occlusal function and it showed failure under high occlusal loads.
3. ME restoration design with VE was the best restorative option for premolar teeth with extensive loss of coronal structure under high occlusal loads.

## Figures and Tables

**Table 1:** Material properties that were assigned to dental tissues, surrounding structure, and restorative materials used [1, 2, 17–20].

	Young's modulus (MPa)	Poisson ratio (*v*)	Characteristic strength (MPa)	Weibull modulus (*m*)	References
Enamel	84100	0.33	42.41	5.53	[1, 17]
Dentin	18600	0.32	44.45	3.35	[1, 17]
Vita Blocks Mark II	71300	0.23	118.65	19.90	[18]
Lava Ultimate	12700	0.45	300.64	10.90	[18]
Vita Enamic	37800	0.24	193.45	18.80	[18]
Spongious Bone	1370	0.3			[2]
Cortical Bone	10700	0.3			[19]
Periodontal ligament	68.9	0.45			[2]
Gutta Percha	0.69	0.45			[20]

**Table 2:** Number of elements and nodes of the models.

Model	Elements	Nodes
Endocrown	300125	56142
Modified endocrown	299891	55885
